# Some initiative in e-waste disposal, management and recycling

**DOI:** 10.4103/0019-5278.64611

**Published:** 2010

**Authors:** Harshal T Pandve

**Affiliations:** Department of Community Medicine, Smt. Kashibai Navale Medical College, Narhe, Pune, Maharashtra, India

Sir,

Electronic and Electrical waste, popularly known as e-waste products, do not decompose or rot away. The disposal of e-waste is a particular problem faced in many regions across the globe. Environment and human health is affected by e-waste. E-waste takes up space in the communities it invades and can be very harmful to humans and animals. E-waste is of concern mainly due to the toxicity and carcinogenicity of some of the substances if processed improperly. As discussed in earlier issues of this journal, there is an urgent need to improve e-waste management covering technological improvement, institutional arrangement, operational plan, protective protocol for workers working in e-waste disposal and, last but not the least, education of general population about this emerging issue posing a threat to the environment as well as public health.[1] To deal with the ever-growing issue of this new type waste various solutions and efforts are underway globally, some of such initiatives are discussed in this article.

Amongst the important initiatives in dealing with e-waste, one is "Plug-in to eCycling". It is a partnership of Environmental Protection Agency (EPA) and consumer electronics manufacturers, retailers, and service providers that offers more opportunities to donate or recycle - to "eCycle" used electronics. ECycling includes recycling and recovers valuable materials from old electronics which can be used to make new products. It also includes reducing greenhouse gas emission, reducing pollution, saving energy and resources by extracting fewer raw materials from the Earth. Safe recycling of outdated electronic items promotes sound management of toxic chemicals such as lead and mercury and helps others.[2] California's Department of Toxic Substances Control (DTSC) requires that used electronics be handled in an environmentally responsible manner. This means that old cellular phones , pagers , telephones and the like cannot be placed in the normal trash. Department of Environmental Health and Safety (EH and S) makes it easy to safely discard these devices. DTSC placed a number of drop-off locations around campus where electronic waste can be discarded. EH and S would collect these items and ensure that they are not sent to landfill for disposal. In fact, majority of these devices would be dismantled and recycled for other uses.[3] Amongst the initiatives taken by developing nations the Nigerian initiative is an important one. To avoid being turned into a dumping ground for e-waste, the Nigerian government has decided to slap duties on old computers imported for spare parts. The decision was made at the federal executive council's meeting in Abuja. There is a growing market for computers and other information and communications technology equipments. But since Nigerians are financially hard-pressed, they mostly depend on affordable second-hand equipments or electronic spare parts. In the absence of proper waste management facilities, burning huge piles of refuse is a common practice in Nigerian cities.[4]

In India also many initiatives regarding e-waste management have gained momentum. Of these, "E-Parisaraa" is a project supported by the Indo-German e-waste initiative. The pilot project to manage e-waste without causing ecological damage has been set up with the backing of the Karnataka State Pollution Control Board in Bangalore city, which would like to see the project replicated in other cities of the country as well. The business model is simple. Most software firms in Bangalore city have agreements with E-Parisaraa to collect their e-waste. E-Parisaraa pays these firms for the e-waste and brings it to their processing facility at Dobbespet in the outskirts of the city. What makes E-Parisaraa different is that unlike the backyard handling of e-waste, there is no melting involved in the sorting. The waste enters the disassembly-line process where it is dismantled and sorted in plastics, rubber and metal sheets. The leftover printed circuit boards and glass items such as tube lights and picture tubes go to the next stage where they are then cut into strips and powdered.[5] A Delhi-based company has launched the country's first helpline dedicated to safe and environment-friendly disposal and recycling of e-waste. Toll-free telephone number is provided to get e-waste picked up from home and recycled.[6] A few e-waste recycling companies providing e-waste management consultancy, electronic and electrical waste recycling are also working in some metropolitan cities.[7,8] In future, the Mumbai Metropolitan Region (MMR) will soon be relieved of the ever growing problem of e-waste. The state government will start the first of its kind plant for scientific recycling of e-waste generated in the region.[9] In Bangalore city installation of e-bins to ensure safe disposal of e-waste generated at government offices in is set to become a reality shortly. Saahas, the Jayanagar-based non-governmental organization (NGO) involved in this pioneering effort, plans to hold campaigns in government offices to create awareness about e-waste and the need to dispose it safely.[10]

To conclude, it is now clear that various initiatives have been started world-wide to deal with the problem of e-waste. Most importantly, research work must be undertaken to explore newer ways to deal with e-waste to make it more eco-friendly; and these initiatives should be continuous and sustainable.
